# Ultrasound-Assisted Multi-Enzyme Extraction for Highly Efficient Extraction of Polysaccharides from *Ulva lactuca*

**DOI:** 10.3390/foods13060891

**Published:** 2024-03-15

**Authors:** Wenqian Wang, Jinbi Li, Fuping Lu, Fufeng Liu

**Affiliations:** Key Laboratory of Industrial Fermentation Microbiology, Ministry of Education, Tianjin Key Laboratory of Industrial Microbiology, College of Biotechnology, Tianjin University of Science and Technology, Tianjin 300457, China; 17853509330@163.com (W.W.); jinbili@mail.tust.edu.cn (J.L.); fplu302@mail.tust.edu.cn (F.L.)

**Keywords:** algal polysaccharides, ultrasound-assisted enzyme extraction, mixture design, response surface methodology, antioxidant activity

## Abstract

*Ulva* polysaccharides present several physiological activities including antiviral, antitumor and anti-plasmodial effects. However, current processing usually results in low yields and high prices, thus lacking commercialization potential. The aim of this study was to develop an efficient method for the extraction of *Ulva* polysaccharides with high biological activity. The effect of cell wall-degrading enzymes including cellulase, hemicellulase, pectinase and protease on *Ulva* polysaccharide extraction was studied by statistical mixing design. Using the most effective enzyme preparations as the basic components, the optimal proportions of the enzyme mixture were determined as follows: cellulase 35.3%, pectinase 34.5%, alkaline protease 30.2%, which increased the polysaccharide yield from 6.43% in the absence of enzymes to 26.68%. Subsequently, through response surface analysis, the optimal conditions were determined: enzyme concentration of 1.5%, enzymatic time of 1.1 h, ultrasonic time of 90 min and enzymatic temperature of 60 °C. Under the optimal extraction conditions, the extraction yield of *Ulva* polysaccharides could be increased to 30.14%. Moreover, extracted polysaccharides exhibit strong antioxidant properties in DPPH, ABTS, hydroxyl radical, superoxide radical and H_2_O_2_-induced cellular damage models. This study laid a solid foundation for the use and development of *Ulva* polysaccharides.

## 1. Introduction

*Ulva lactuca* (*U. lactuca*) is a large edible green alga that is widely distributed in coastal areas worldwide [1]. The whole *U. lactuca* is widely used to treat various inflammatory diseases and hypertension [2]. Studies have shown that the main functional component of *U. lactuca* is the polysaccharide present in the cell wall [3], which has rich biological properties, such as moisturizing, antiviral and immunoregulatory effects, so it has broad application prospects in biomaterials [4], disease treatment [5] and functional foods [6]. Recently, researchers have been interested in using *Ulva* polysaccharides to produce functional foods and dietary supplements to alleviate various metabolic and chronic diseases. For example, Chen et al. [7] found that *Ulva* polysaccharides could prevent age-related diabetes by influencing intestinal flora, and Yu et al. [8] showed that *Ulva* polysaccharides could promote lipid metabolism and regulate lipid levels. At present, the high price due to low extraction efficiency is one of the main barriers limiting the popularization and application of *Ulva* polysaccharides. Therefore, more and more attention has been paid to the development of environmentally friendly and efficient extraction methods to obtain *Ulva* polysaccharides with high biological activity.

Extraction methods have had a major impact on the yield and activity of polysaccharides [9]. Recently, innovative polysaccharide extraction techniques have emerged, such as microwave-assisted extraction, ultrasound-assisted extraction (UAE) and enzyme-assisted extraction (EAE) [10]. EAE is considered to be a potential method for polysaccharide extraction with advantages of a low cost, mild operating conditions and maintaining high biological activity of the extract [11]. It has been found that cellulase can effectively improve the extraction rate of Ulva polysaccharides [12]. In addition, the research of Rani et al. [13] and Cristina et al. [14] showed that the combination of several enzymes can effectively destroy the cell wall and facilitate the extraction of active ingredients. UAE, which is based on acoustic cavitation facilitation, is beneficial for increasing sample–solvent contact and is widely used for polysaccharide extraction [15]. Ultrasound-assisted enzymatic extraction (UAEE) for synergistic degradation of plant cell walls to accelerate polysaccharide release is an emerging and efficient extraction technology. To date, UAEE has been used to extract various polysaccharides such as *Ecklonia cava* [16] and *Scutellaria baicalensis* root [17] polysaccharides.

The aim of this study was to develop a new extraction process of *Ulva* polysaccharides to improve production efficiency and biological activity. Therefore, the enzymes suitable for *U. lactuca* cell wall degradation were screened by the UAEE method using inexpensive commercial enzyme preparations. The cell wall-degrading mixed-enzyme preparation was prepared by simplex lattice mixture design to synergistically promote the release of *Ulva* polysaccharides. Subsequently, single-factor experiments and the Box–Behnken design (BBD) were used to optimize the extraction conditions to maximize the extraction yield of *Ulva* polysaccharides. Furthermore, the antioxidant activity was evaluated by measuring the efficiency of the extracts in scavenging 1, 1-diphenyl-2-picrylhydrazine (DPPH), hydroxyl, 2, 2′-azinobis-(3-ethylbenzothiazoline-6-sulfonic acid) (ABTS) and superoxide free radicals. Finally, the oxidative damage model of SH-SY5Y cells induced by H_2_O_2_ was used to investigate the protective effect of the extracts on cells under oxidative stress. It provided a valuable theoretical basis for the efficient extraction and pharmacological application of *Ulva* polysaccharides.

## 2. Materials and Methods

### 2.1. Materials

*U. lactuca* powder was purchased from Haixingyuan Biotechnology Co., Ltd. (Qingdao, China). Cellulase (CEL), pectinase (PEC), papain (PAP) and neutral protease (NP) were obtained from Yuanye Biotechnology Co., Ltd. (Shanghai, China). Alcalase (ALC) was from Solarbio (Beijing, China). Xylanase (AXC) and β-mannase (GMA) were purchased from Macklin (Shanghai, China). DPPH, ABTS and pyrogallol were from Soleibao Technology Co., LTD. (Beijing, China). Cell culture reagents were from Cytiva (Middletown, DE, USA). 2,7-Dichlorodihydrofluorescein diacetate (DCFH-DA) was obtained from Beyotime Biotechnology Co., Ltd. (Shanghai, China).

### 2.2. Ultrasound-Assisted Enzymatic Polysaccharide Extraction

A process for the extraction of *Ulva* polysaccharides by UAEE was investigated using the treatment described by Li et al. [18]. Briefly, with slight modifications, *U. lactuca* powder was mixed with water (1:30, *w*/*v*) and the enzyme preparation was added. The mixture was stirred by continuous magnetic force in a water bath at 50 °C for 2 h, and then underwent ultrasonic treatment for 60 min. The solids and liquids were separated by centrifugation (8000 rpm, 10 min). The supernatant was retained, concentrated under reduced pressure, ethanol was added at a ratio of 1:4 (*v*/*v*) and the polysaccharide was recovered overnight at 4 °C. Subsequently, the mass of crude polysaccharides was determined by gravimetry after centrifugation, retention precipitation and lyophilization. The enzyme dosage was 10 mg/g for both the screening and mixture design experiments. The content of polysaccharides was determined by the phenol–sulfuric acid method [19], and the extraction yield of polysaccharides was calculated:

(1) $$Y=\frac{C\times M}{M_{0}}\times 100$$

where *Y* is the polysaccharide extraction rate of *U. lactuca* (%), *C* is the content of polysaccharides in the extract (%); and *M* and *M_0_* are the dry weight of the extract and *U. lactuca*, respectively.

### 2.3. Scanning Electron Microscopy (SEM) Assay

A small amount of sample was uniformly distributed on a metal plate using a JS-1600 ion sputterer (Sun Yi Experimental Equipment Co., Ltd., Nanjing, China) and a thin layer of gold was sprayed on it. Micrographs of the algae were taken using an SEM (SU1510, Hitachi) at a scale of 50 μm and an acceleration voltage of 15 Kv.

### 2.4. Mixture Design

An augmented simplex centroid design was used to study the effect of mixed enzymes on *Ulva* polysaccharide extraction [20], consisting of a four-factor, three-level simplex lattice design with replication vertices and centers, including 20 mixture schemes. The *Ulva* polysaccharide extraction yield (*Y_pol_*, %) was used as the response variable by varying the concentration of each component enzyme and keeping the total concentration of the enzyme preparation constant. The order of the experiments was randomized; details of the experimental design are provided in Table 1. The experimental design and analysis of the results were performed using Design-Expert 13.0 software.

### 2.5. Optimization of Polysaccharide Extraction Conditions

The Box–Behnken design–response surface methodology (BBD-RSM) was used to optimize the UAEE procedure to improve the extraction efficiency of *Ulva* polysaccharides [21]. Enzyme concentration, enzymatic time, ultrasonic time and enzymatic temperature were selected as the main factors for the optimization of the UAEE process [22]. Table 2 shows the encoding and scope of the factors. Design-Expert 13.0 was used to design the BBD project with four independent variables at three levels to optimize the UAEE process. As shown in Table 3, the scheme was composed of 5 center points and 24 factor points, for a total of 29 runs, with *Y_pol_* as the response value of the design experiment. The resulting data were fitted with a quadratic polynomial model [23]:

(2) $$Y=b_{0}+\sum_{i=1}^{4}b_{i}X_{i}+\sum_{i=1}^{4}b_{ii}{X_{i}}^{2}+\sum_{i>1}^{4}\sum_{j}^{4}b_{ij}X_{i}X_{j}$$

where Y is the polysaccharide extraction rate of *U. lactuca* (%), *b*_0_, *b_i_*, *b_ii_* and *b_ij_* are the regression coefficients and *X_i_* and *X_j_* are the coded independent factors.

### 2.6. Antioxidant Activity Assay

#### 2.6.1. Scavenging Effect of DPPH Radicals

The extracted crude polysaccharide was purified on cellulose DEAE and Sephadex G-100 columns, and its antioxidant activity was determined [24]. In total, 2 mM DPPH–ethanol solution was mixed with the samples in a 1:1 volume ratio, protected from light for 30 min, and the absorbance at 517 nm was measured using a microplate reader (SpectraMax i3X, Molecular Devices, Shanghai, China) [25]. Deionized water was used as a control and ethanol as a blank. The calculation formula was

(3) $$Scavenging\;effect\;\left(\%\right)=\left[\frac{A_{b}-A_{u}}{A_{b}}\right]\times 100$$

where *A_b_* and *A_u_* were the absorbance of blank control and *Ulva* polysaccharides, respectively.

#### 2.6.2. Scavenging Effect of Hydroxyl Radical

The hydroxyl radical scavenging activity of polysaccharides was determined by the Fenton method with slight modifications [26]. Briefly, 1 mL samples were added to 1 mL FeSO_4_ (9 mM), 1 mL salicylic acid–ethanol solution (9 mM) and 1 mL H_2_O_2_ (8.8 mM) and incubated in a 37 °C water bath for 30 min. Considering the absorbance of the samples themselves and other solutions, bidistilled water instead of hydrogen peroxide was used as background and bidistilled water instead of the sample solution was used as negative control. The absorption value of the mixture was read at 562 nm [27], and the calculation formula was

(4) $$Scavenging\;effect\;\left(\%\right)=\left[1-\frac{A_{s}-A_{b}}{A}\right]\times 100$$

where *A_s_*, *A_b_* and *A* are the absorbance of the sample, sample background and negative control without sample, respectively.

#### 2.6.3. Scavenging Effect of ABTS Radical

In total, 2 mL ABTS (7.4 mM) was mixed with 2 mL K_2_S_2_O_8_ (2.6 mM), reacted at 25 °C for 12 h protected from light and diluted to an ABTS working liquid with PBS buffer (pH 7.4) [28]. Then, 50 μL of the extract was mixed with 3 mL ABTS working solution and reacted for 6 min, protected from light. Here, 50 μL of distilled water was used as a blank group instead of the extract, and 50 μL of the extract was mixed with 3 mL of 10 mM PBS buffer (pH 7.4) as a control [29]. The degradation rate of ABTS radicals by copolymer was calculated as follows:

(5) $$Scavenging\;effect\;\left(\%\right)=\left[A_{b}-\left(A_{u}-A_{c}\right)\right]\times \frac{100}{A_{b}}$$

where *A_b_*, *A_u_* and *A_c_* are the absorbance of the blank group, detection group and control group, respectively.

#### 2.6.4. Scavenging Effect of Superoxide Anion Radical

In total, 5 mL Tris-HCl buffer (50 mM, pH 8.2) was added to 4 mL of the samples in a water bath at 25 °C for 20 min. Then, 1 mL of pyrogallol (3 mM) was added and reacted at 25 °C for 5 min. After that, 1 mL of HCl (10 mM) was used to terminate the reaction and the absorption values at 320 nm were measured. In the blank group, the sample was replaced with deionized water:

(6) $$Scavenging\;effect\;\left(\%\right)=\left[\frac{A_{b}-A_{u}}{A_{b}}\right]\times 100$$

where *A_b_* and *A_u_* are the absorbance of the blank group and detection group, respectively.

### 2.7. Cellular Experiments

#### 2.7.1. Cell Culture and Treatment

Human myeloid neuroblastoma cells SH-SY5Y were cultured in DMEM and supplemented with 10% fetal bovine serum and 1% penicillin/streptomycin. The cell incubator was maintained at 5% CO_2_, 37 °C and constant humidity. Fresh medium was replaced every two days, and normal cells at the logarithmic phase were retained for subsequent experiments.

#### 2.7.2. MTT Assay

Cell viability was measured by the MTT assay with minor modifications [30]. The logarithmic phase SH-SY5Y cells (5 × 10^4^/mL) were inoculated into 96-well plates and placed in an incubator for 24 h. Then, *Ulva* polysaccharides with gradient concentrations (25, 50, 100 and 200 μg/mL) were treated for 24 h. Subsequently, the cells were treated with H_2_O_2_ (250 μM) for 24 h to induce oxidative stress, and the H_2_O_2_ in the control group was replaced with the medium [31]. Finally, the medium was replaced with (500 µg/mL) MTT solution and treated for 4 h. The blue-violet formazan crystal was dissolved with DMSO, and the absorbance at 570 nm was measured. The cell viability of the control group was defined as 100% for data normalization.

#### 2.7.3. Reactive Oxygen Species (ROS) Production Assay

The ROS levels in the SH-SY5Y cells were measured according to the described procedure, with some modifications [32]. SH-SY5Y cells (2 × 10^5^/mL) were inoculated into 6-well plates and cultured for 24 h. After treatment with *Ulva* polysaccharides and H_2_O_2_ (as described in Section 2.7.2), the cells were washed three times with PBS. The fluorescent probe DCFH-DA was dilute them to 10 µM with serum-free cell medium, and then the SH-SY5Y cells were treated with it for 30 min at 37 °C in the dark. Serum-free cell culture medium was then used to remove the fluorescent probe. The fluorescence signal was measured using a microplate reader (SpectraMax i3X, Molecular Devices, Shanghai, China), λ_ex_ = 480 nm, λ_em_ = 520 nm.

### 2.8. Statistical Analysis

All experiments were performed in triplicate and the data are represented as mean ± SD. The GraphPad Prism 8.0 software was used for statistical analysis and graphing, and *p* < 0.05 was considered statistically significant.

## 3. Results and Discussion

### 3.1. Determining the Optimal Combination of Enzymes

**Figure 1:**
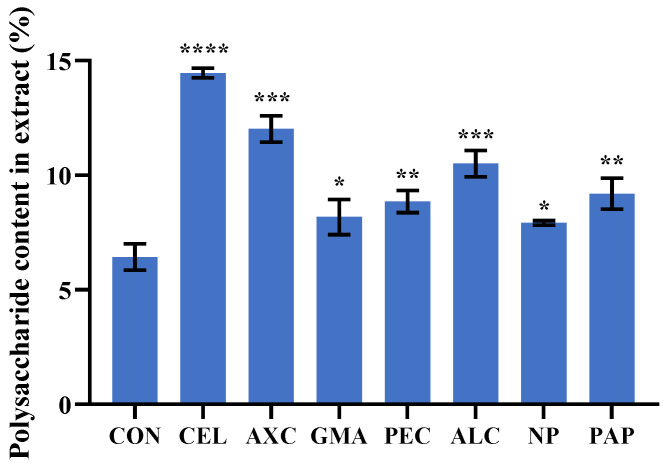
The results of the screening of the enzyme preparations. CON represents the control group without added enzymes, compared to the control, * *p* < 0.05, ** *p* < 0.01, *** *p* < 0.001 and **** *p* < 0.0001.

Enzyme preparations suitable for *Ulva* polysaccharide extraction were screened according to the characteristics of *U. lactuca* cell walls (Table 4). *U. lactuca* has a bilayer cell wall structure consisting mainly of cellulose, hemicellulose and pectin [33]. Therefore, CEL, hemicellulase (AXC and GMA) and PEC were included in the screening set. In addition, the *U. lactuca* cell wall contains a small amount of protein. Kevin et al. [34] showed that proteases could effectively improve the extraction yield of *Ulva* polysaccharides, so ALC, PAP and NP were included. Polysaccharide extraction was carried out under the optimal conditions for each enzyme, and the screening experiment results are shown in Figure 1. The extraction yield of *Ulva* polysaccharides was 6.43% without any enzyme preparation. In comparison, all the enzyme preparations used in the experiment increased the extraction rate of polysaccharides. For example, CEL, AXC, GMA, PEC, ALC, NP and PAP treatments could increase the polysaccharide content in extracts to 14.46%, 12.02%, 8.18%, 8.85%, 10.51%, 7.91% and 9.19%, respectively (Figure 1). This indicates that enzyme treatment may be an effective way to break through the cell wall, an important barrier for polysaccharide extraction, thus facilitating the rapid release of polysaccharides from the cells. Specifically, CEL, AXC, PEC and ALC had a higher polysaccharide extraction rate than the same kind enzyme preparations (Figure 1), so they could be used as the basic components of the complex enzyme preparations.

**Figure 2:**
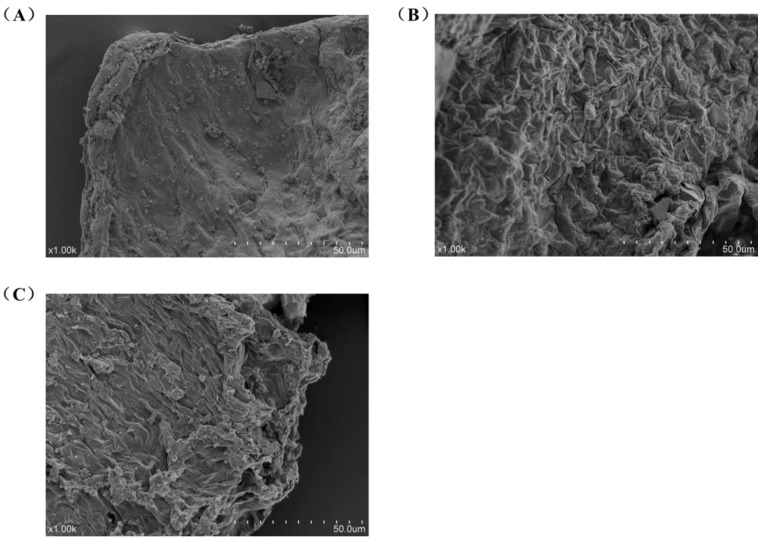
SEM micrographs of the surface structure of *U. lactuca* samples (1000×): (**A**) untreated, (**B**) ultrasound-assisted hot water treatment and (**C**) ultrasound-assisted cellulase treatment.

Further, the microstructure of *U. lactuca* was observed by SEM to determine the beneficial effects of enzymes on cell wall destruction. As shown in Figure 2, the intact surface of the untreated sample tissue was smooth (Figure 2A), while the ultrasonic-assisted hot water treatment caused tissue damage and wrinkles (Figure 2B), resulting in the dissolution of some polysaccharides. As expected, the addition of CEL significantly disrupted the cell walls and membranes, resulting in a lamellar structure and a large number of gaps and cavities (Figure 2C), which contributed to the release of polysaccharides. The results were consistent with the screening experiment, indicating that the enzyme treatment caused the disruption or loosening of the cell wall, which facilitated the infiltration of solvent molecules into the cells and the enhanced polysaccharide extraction.

Considering the complex composition of *U. lactuca*’s cell wall, the degrading effect of a single enzyme was limited, as the complex action of multiple enzymes is conducive to the destruction of the cell wall [35]. Therefore, CEL, AXC, PEC and ALC were used as the basic components of complex enzyme preparations and coupled. The experiments were performed according to the simplex lattice design, with each point corresponding to a specific mixture composition, and the obtained results are shown in Table 1. The Scheffé canonical polynomial model (special cubic) was used for regression analysis. The variation in *Y_pol_* values corresponding to different ratios of the enzyme was fitted using Design-Expert to obtain the reduced model for CEL (*x*_1_)-AXC (*x*_2_)-PEC (*x*_3_)-ALC (*x*_4_):

(7) $$Y_{pol}=13.70x_{1}+13.04x_{2}+8.35x_{3}+10.92x_{4}+11.19x_{1}x_{2}+45.82x_{1}x_{3}+0.30x_{1}x_{4}+30.46x_{2}x_{3}-0.64x_{2}x_{4}\;+7.69x_{3}x_{4}+87.00x_{1}x_{2}x_{3}+390.87x_{1}x_{2}x_{4}-129.31x_{1}x_{3}x_{4}+213.88x_{2}x_{3}x_{4}$$

According to the analysis of variance (Table 5), Prob (P) > *F* < 0.001, indicating that the modified equation had a high fitting accuracy. The correlation coefficient R^2^ = 0.9842 and adjusted R^2^ = 0.9500, indicating a good fit of the model. Furthermore, no significant deviation from the basic assumptions of ANOVA was found, and the *p*-value of the lack of fit was 0.9690, indicating that the model had high stability (Table 5). In addition, CV = 7.36% < 10%, indicating high confidence in the experiment. In summary, the fitted regression equation was consistent with the test principle and had good adaptability, which can be used for subsequent optimization designs.

**Figure 3:**
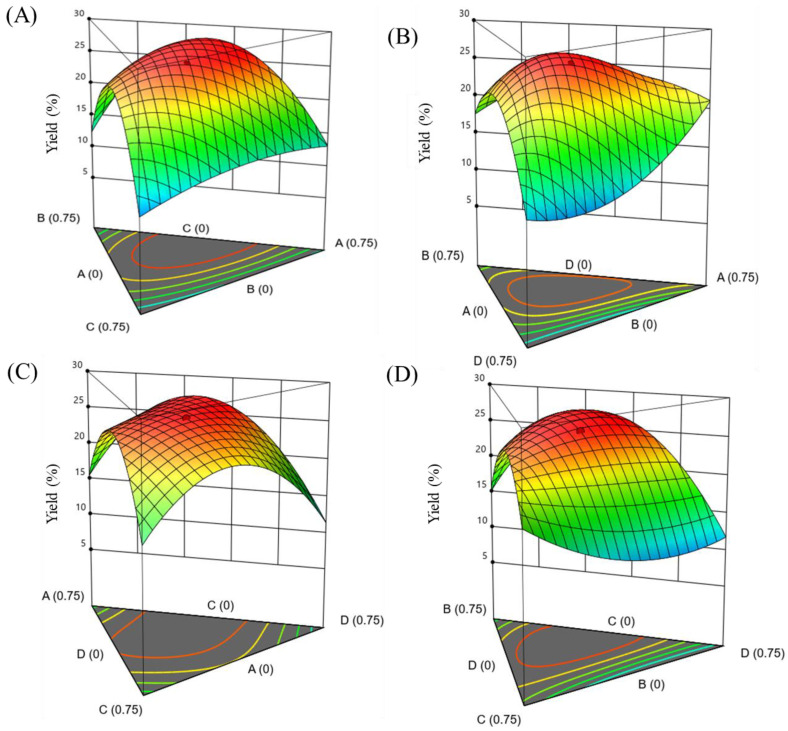
Three-dimensional response surface plots of *Ulva* polysaccharide extraction yield (%) calculated from Equation (7). The weight fraction of the fourth mixture component was set to 0.75. (**A**): CEL; (**B**): AXC; (**C**): PEC; (**D**): ALC.

The coefficient of the positive term in the fitting equation represents the positive correlation between the factor and the response value, and the larger the coefficient, the stronger the correlation [36]. According to Equation (7), the single-enzyme preparation had a beneficial effect on the dissolution of polysaccharides, and the influence of the four enzyme preparations on the polysaccharide extraction was in the following order: CEL > AXC > ALC > PEC, which was consistent with the results of the screening experiments (Figure 1). Three-dimensional response surfaces and two-dimensional contours could show the interaction between the different factors, for example, surface convexity reflects synergistic effects and surface concavity vice versa. As shown in Figure 3 and Figure S1, ternary mixed-enzyme preparations have both synergistic and antagonistic effects at the same time. For example, when CEL-PEC-ALC were used together, they showed antagonism; the mixed-enzyme preparations of CEL-AXC-PEC, CEL-AXC-ALC and AXC-PEC-ALC showed synergistic effects and CEL-AXC-ALC had the greatest synergistic effect. For this purpose, numerical calculations were performed by maximizing the corresponding variables in the equation, and the following the numerical calculation results were obtained: *x*_1_ = 0.353, *x*_2_ = 0.345, *x*_3_ = 0.302 and *Y_lop_* = 28.34%. Validation experiments were performed with the above mixed-enzyme preparations and the resulting polysaccharide content of 26.68% differed from the predicted value by <2%.

### 3.2. Optimization of Ultrasound-Assisted Enzymatic Extraction

The BBD was used to optimize the UAEE process for the extraction of *Ulva* polysaccharides. With the extraction rate of *Ulva* polysaccharides as the response value, the relationship between the predicted response value and various factors can be expressed by a polynomial equation, as follows:

(8) $$Y_{pol}=32.98-1.13X_{1}+0.94X_{2}+1.81X_{3}-0.76X_{4}+1.24X_{1}X_{2}+1.73X_{1}X_{3}+0.19X_{1}X_{4}+1.01X_{2}X_{3}\;+0.28X_{2}X_{4}-0.08X_{3}X_{4}-3.69X_{1}^{2}-4.01X_{2}^{2}-2.08X_{3}^{2}+3.21X_{4}^{2}$$

The statistical analysis results of this model are shown in Table 6, with *p* < 0.0001 and the *F*-value (24.17) indicating that the fitted polynomial could well characterize the relationship between the parameters [37]. The *p*-value of the lack of fit was 0.6440, confirming the validity of the experimental model and that unknown factors had little effect. Furthermore, predictions were made for the response values of the regression Equation (8): the correlation coefficient R^2^ = 0.9603, which means that the model has a 96.03% agreement with the actual test fit. In addition, the coefficient variability of the model, CV = 3.34%, indicates a high degree of experimental reproducibility. In general, the fitted model was an ideal model which was sufficient to cover the experimental design area and could be used in subsequent experiments.

**Figure 4:**
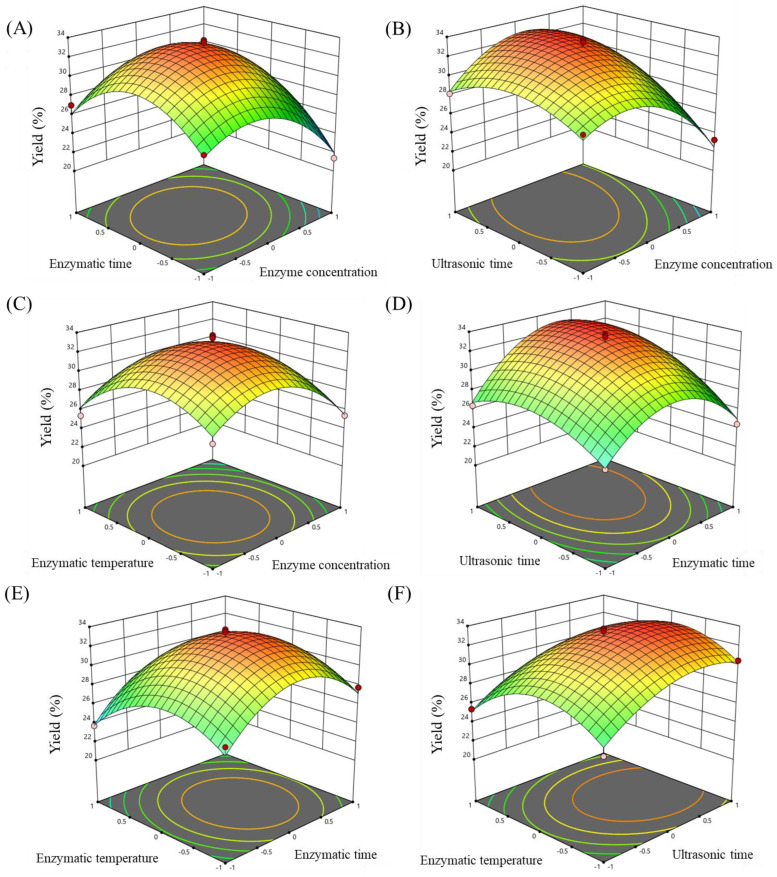
(**A**–**F**) Three−dimensional response surface plots of *Ulva* polysaccharide extraction yield (%)influenced by different enzyme concentration, enzymatic time, ultrasonic time and enzymatic temperature.

The response surface diagram more intuitively reflected the influence of two factors (other variables fixed at 0 level) on the extraction yield of *Ulva* polysaccharides. The greater the slope of the 3D surface, the stronger the effect of the independent variables on the extraction rate [38]. Moreover, the ellipticity of the contour lines shows whether the factor has a significant effect on the response value [39]. As shown in Figure 4 and Figure S2, the effects of enzyme concentration and ultrasonic time on polysaccharide yield were more significant compared to enzymatic time and enzymatic temperature. Furthermore, the interaction of *X*_1_*X*_2_, *X*_1_*X*_3_ and *X*_2_*X*_3_ was significant and the interaction effect of *X*_1_*X*_4_, *X*_2_*X*_4_ and *X*_3_*X*_4_ was not significant. The analysis results of the RSM were in good agreement with the results of the analysis of variance of the regression model (Table 6), which proved that the test results were highly representative. According to the BBD results, the optimal technological parameters of *Ulva* polysaccharide extraction were obtained: an enzyme concentration of 1.49%, enzymatic time of 1.08 h, ultrasonic time of 89.42 min and enzymatic temperature of 58.82 °C. According to the actual situation, the modified parameters were 1.5% enzyme concentration, 1.1 h enzymatic time, 90 min ultrasonic time and 60 °C enzymatic temperature for experimental verification. The polysaccharide yield was 30.14%, and the relative error of the predicted value was 0.41%. This shows that the predicted values fit well with the actual values, and the model has good practical reference significance.

Compared with the reported extraction methods of *Ulva* polysaccharides, the optimized UAEE process can not only significantly improve the extraction rate of polysaccharides but also save energy consumption and time. For example, Xu et al. [40] obtained a 21.96% extraction yield of *Ulva* polysaccharides by using a 90 °C hot water treatment for 4 h and obtained a 20.22% extraction yield by using cellulase to promote polysaccharide dissolution. Lü et al. [41] obtained 27.75% of *Ulva* polysaccharides by protease-assisted extraction. Although the extraction yield of acid and alkaline extraction was higher, for example, 33.30% polysaccharides could be obtained by using an alkaline solution at 90 °C for 2 h [42] and up to 38.35% polysaccharides could be produced by acid extraction at 80 °C for 24 h [43], acid and alkaline extraction could break the glycosidic bond and change the polysaccharide configuration, and special reactions during extraction may produce by-products. On the other hand, in order to prevent environmental pollution, the liquid after acid and alkaline extraction should be pH neutralized, and the post-processing is more complicated. Therefore, acid and alkaline extraction of polysaccharides is not considered a promising method.

### 3.3. Ulva Polysaccharide Extract Effectively Scavenges Free Radicals

**Figure 5:**
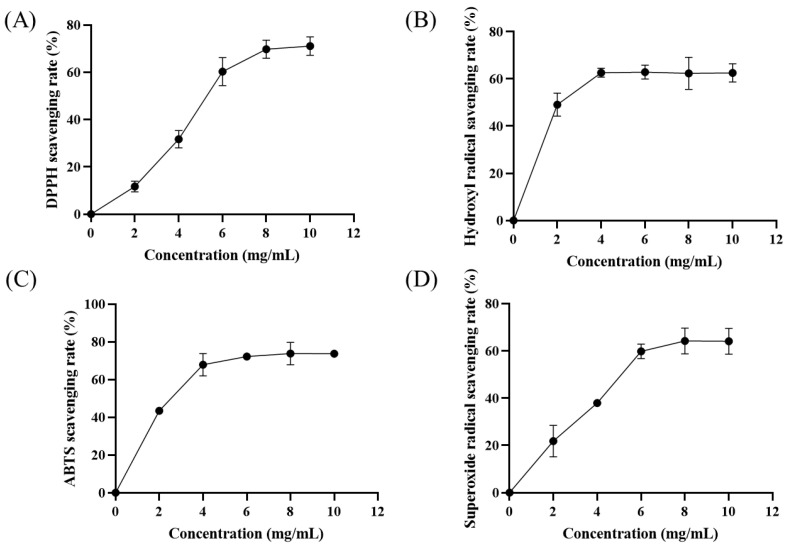
The scavenging effect of *Ulva* polysaccharides on (**A**) DPPH, (**B**) hydroxyl, (**C**) ABTS and (**D**) superoxide anion radicals.

In molecular biology, high levels of free radicals have been closely linked to the onset of degenerative processes. They could enhance oxidative stress, leading to inadequate cell function, aging and even disease [44]. Therefore, the antioxidant capacity of polysaccharides is an important index to evaluate their biological activity. DPPH scavenging ability detection is a simple, rapid and reliable method for the study of antioxidant properties of natural products. As shown in Figure 5A, in the range of 0~6.0 mg/mL, the scavenging activity of DPPH free radicals was significantly enhanced with the increase in the concentration of *Ulva* polysaccharides. A total of 8.0 mg/mL *Ulva* polysaccharides can effectively remove 69.80% of DPPH free radicals. Subsequently, it was calculated that the sample concentration required to scavenge half of the free radicals (SC_50_) was 5.46 mg/mL. Compared with other methods, the *Ulva* polysaccharides extracted by the UAEE method had better DPPH scavenging activity. For example, the DPPH scavenging SC_50_ values of *Ulva* polysaccharides obtained by enzyme-assisted and ultrasonic-enzyme-assisted extraction were 6.52 and 9.90 mg/mL, respectively [45].

The hydroxyl radical is the most harmful free radical for an organism, and it is capable of having a free radical chain reaction with almost any biological macromolecule in living cells [46]. As shown in Figure 5B, *Ulva* polysaccharide extracts of different concentrations had scavenging effects on the hydroxyl radical. However, when the extract concentration was higher than 4 mg/mL, the scavenging effect was not significantly improved. Compared with *Ulva* polysaccharides obtained by pressurized water-assisted extraction, the polysaccharide extracted with UAEE showed a better hydroxyl radical scavenging effect. For example, the hydroxyl radical scavenging rates of 2 mg/mL UAEE-extracted and pressurized water-assisted-extracted *Ulva* polysaccharides were 49.12% and 45% [3], respectively. In addition, previous studies have shown that the hydroxyl radical scavenging activity is related to the molecular weight of the compound [43]. The high hydroxyl radical scavenging activity of *Ulva* polysaccharides suggests that they have a lower molecular weight, which affects the solubility and viscosity of the polysaccharide, thus improving the antioxidant activity.

The ABTS free radical scavenging method is widely used to determine the total antioxidant capacity of biological samples [47]. The ABTS scavenging activity of *Ulva* polysaccharides is shown in Figure 5C. As expected, increasing the concentration of *Ulva* polysaccharides resulted in an increase in ABTS scavenging. When the concentration of *Ulva* polysaccharides increased from 0 mg/mL to 4 mg/mL, the scavenging rate of ABTS radical increased from 0% to 67.85%. In the range of 6.0~10.0 mg/mL, the ABTS free radical scavenging rate of *Ulva* polysaccharides remained about 73%. Moreover, the highest ABTS scavenging rate of *Ulva* polysaccharides extracted by UAEE was 73.81%, which was higher than those extracted by the hot water (68.06%), alkali (61.01%) and acid (71.87%) methods [43].

Superoxide free radicals play an important role in the oxidative and reductive metabolism of cells, can participate in many physiological activities such as cell proliferation and apoptosis, and are closely related to body aging and disease [48]. Therefore, the scavenging activity of superoxide free radicals is very important to antioxidant work. As shown in Figure 5D, the scavenging effect of 0~8 mg/mL *Ulva* polysaccharides on superoxide free radicals was concentration-dependent. Among them, 8 mg/mL *Ulva* polysaccharides could remove 64.26% of superoxide free radicals. Subsequently, with the increase in polysaccharide concentration, the free radical scavenging rate did not increase significantly, and the inhibitory concentration (IC_50_) was 5.32 mg/mL. For polysaccharides with special conformation, the hydrogen in the oxygen–hydrogen bond is easily released, thus stabilizing the superoxide free radicals [49]. The mechanism of polysaccharide removal of superoxide free radicals may be related to the dissociation energy of the oxygen–hydrogen bond.

### 3.4. Ulva Polysaccharides Protect SH-SY5Y Cell Damage Induced by H_2_O_2_

**Figure 6:**
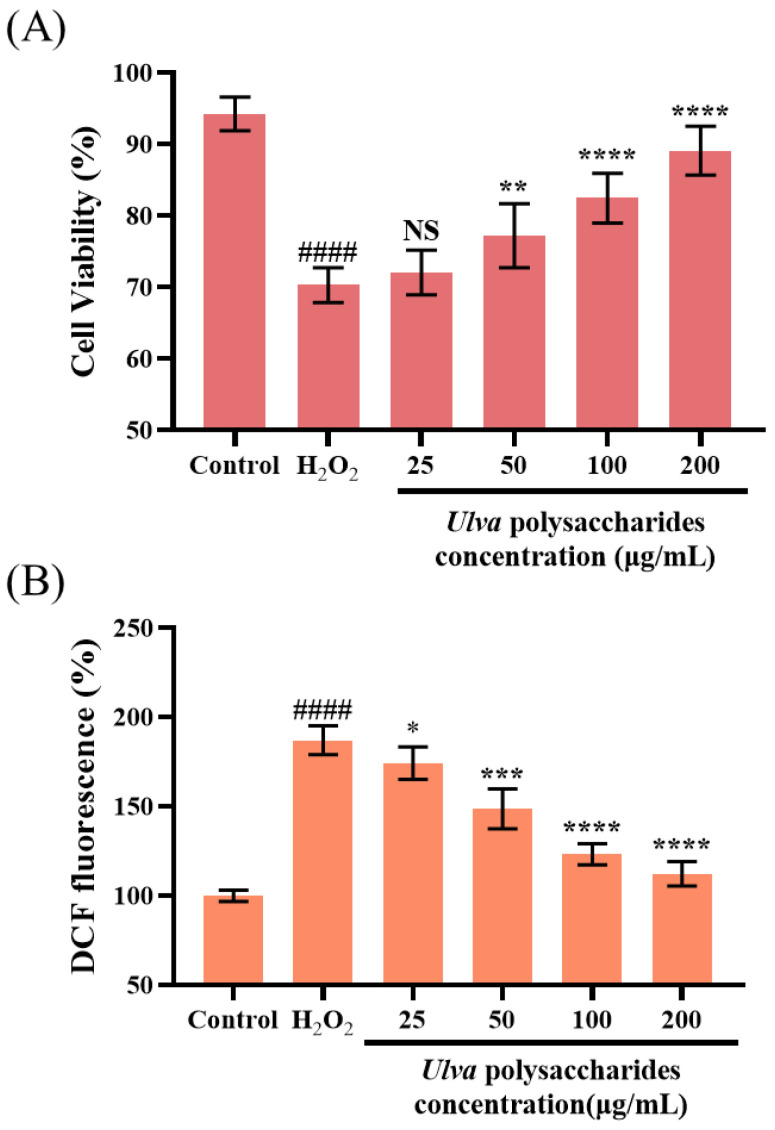
Protective effect of *Ulva* polysaccharides on SH-SY5Y cells induced by H_2_O_2_. (**A**) *Ulva* polysaccharides attenuated H_2_O_2_-induced cytotoxicity. (**B**) *Ulva* polysaccharides inhibited the increase in ROS levels induced by H_2_O_2_. Data are expressed as means ± SD (*n* = 5). Compared with the control group, #### *p* < 0.0001. Compared with the group treated with H_2_O_2_ alone, NS, not significant; * *p* < 0.05, ** *p* < 0.01, *** *p* < 0.001 and **** *p* < 0.0001.

Based on the study of cell-free systems, we further investigated the protective effect of *Ulva* polysaccharide pretreatment on oxidative stress in intact cell models. H_2_O_2_ destroyed the protein structure through oxidative reaction, triggered mitochondrial dysfunction and led to apoptosis, and was a common compound used to establish cell models of oxidative damage [50]. An MTT assay was used to investigate the mitigating effect of *Ulva* polysaccharides on oxidative damage in cells, and the cell viability of the blank control group was defined as 100%. As shown in Figure 6A, as expected, 250 μM H_2_O_2_ reduced cell viability to 70.31%, indicating severe cell damage induced by H_2_O_2_. *Ulva* polysaccharide pretreatment was effective in alleviating the oxidative damage caused by H_2_O_2_ in a dose-dependent manner, specifically, 25, 50, 100 and 200 μg/mL *Ulva* polysaccharide treatment increased the cell activity to 72.05%, 77.20%, 82.47% and 89.11%, respectively.

ROS can induce oxidative stress and lead to apoptosis by regulating active transcription factors [51]. The antioxidant activity of *Ulva* polysaccharides was evaluated by detecting ROS levels. H_2_O_2_-stimulated SH-SY5Y showed significantly higher ROS levels than normal cultured cells, indicating oxidative stress. However, the H_2_O_2_-induced elevation of ROS levels gradually decreased to normal levels with the increase in *Ulva* polysaccharides. In particular, 200 μg/mL of *Ulva* polysaccharides could reduce the ROS to 112.26% (Figure 6B). Consistent with the results of Zhang et al. [52], pretreatment with antioxidant substances could alleviate cytotoxicity and inhibit ROS production to play a cytoprotective role in oxidatively stressed cells.

## 4. Conclusions

In this study, *Ulva* polysaccharides were extracted by ultrasound-assisted enzyme preparation. Enzyme mixtures with improved cell wall destruction activity were prepared by using the augmented simplex lattice design. The mixed-enzyme preparation composed of 35.3% CEL, 34.5% AXC and 30.2% ALC could effectively destroy the cell wall and increase the extraction rate of *Ulva* polysaccharides by 20.25%. Subsequently, the RSM-BBD was used to optimize the extraction conditions to further improve the recovery of polysaccharides. The optimum process parameters of polysaccharide extraction were as follows: enzyme concentration of 1.5%, enzymatic time of 1.1 h, ultrasonic time of 90 min and enzymatic temperature of 60 °C. Under these conditions, the yield of *Ulva* polysaccharides was 30.14%. Compared with the traditional experimental method, the optimized UAEE not only had the advantages of low energy consumption, easy industrialization integration and safety but also greatly improved the extraction yield of *Ulva* polysaccharides.

In addition, *Ulva* polysaccharides extracted by UAEE showed good antioxidant activity in vitro. In cell-free systems, 6 mg/mL of the polysaccharide could effectively remove 60.33% DPPH, 62.90% hydroxyl, 72.23% ABTS and 59.81% superoxide free radicals. Furthermore, *Ulva* polysaccharides could significantly reverse the increase in ROS levels induced by hydrogen peroxide in SH-SY5Y cells and improve cell viability. For example, 200 μg/mL of *Ulva* polysaccharides reduced ROS to 112.26% and restored cell viability to 89.11%. In general, the multi-enzyme synergistic ultrasonic extraction method not only improved the extraction rate of *Ulva* polysaccharides, which is expected to promote the practical application of *Ulva* polysaccharides in biomedicine and food, but the method preserved the biological activity of *Ulva* polysaccharides to a great extent; the prepared *Ulva* polysaccharides have good antioxidant activity in vitro and are a bioactive substance worthy of further research and development.

## Figures and Tables

**Table 1:** Mixture design layout and determination of *Ulva* polysaccharide extraction yield (*Y_pol_*).

Standard Order	Running Order	*x*_1_	*x*_2_	*x*_3_	*x*_4_	*Y_pol_* (%)
1	14	1	0	0	0	13.22
2	2	0	1	0	0	13.53
3	20	0	0	1	0	11.73
4	17	0	0	0	1	8.92
5	19	0.5	0.5	0	0	14.61
6	8	0.5	0	0.5	0	12.39
7	16	0.5	0	0	0.5	22.48
8	9	0	0.5	0.5	0	11.82
9	4	0	0.5	0	0.5	18.31
10	5	0	0	0.5	0.5	11.56
11	13	0.625	0.125	0.125	0.125	21.47
12	6	0.125	0.625	0.125	0.125	22.80
13	11	0.125	0.125	0.625	0.125	17.95
14	1	0.125	0.125	0.125	0.625	19.08
15	10	0.25	0.25	0.25	0.25	26.25
16	15	1	0	0	0	14.18
17	12	0	1	0	0	12.55
18	18	0	0	1	0	10.12
19	7	0	0	0	1	7.78
20	3	0.5	0.5	0	0	17.72

*x*_1_: cellulase; *x*_2_: xylanase; *x*_3_: pectinase; *x*_4_: alcalase.

**Table 2:** The independent variable levels used in BBD and their symbols.

Independent Variables	Symbol	Levels		
		−1	0	1
Enzyme concentration (%)	*X*_1_	1.0	1.5	2.0
Enzymatic time (h)	*X*_2_	0.5	1.0	1.5
Ultrasonic time (min)	*X*_3_	60	80	100
Enzymatic temperature (°C)	*X*_4_	50	60	70

**Table 3:** BBD for the optimization of *Ulva* polysaccharide extraction conditions and the results of polysaccharide extraction yield (*Y_po_*_l_).

Standard Order	Running Order	*X*_1_	*X*_2_	*X*_3_	*X*_4_	*Y_pol_* (%)
1	25	−1	−1	0	0	23.97
2	13	1	−1	0	0	18.40
3	8	−1	1	0	0	24.05
4	17	1	1	0	0	23.4
5	16	0	0	−1	−1	22.72
6	29	0	0	1	−1	27.49
7	11	0	0	−1	1	22.42
8	22	0	0	1	1	26.88
9	20	−1	0	0	−1	24.52
10	28	1	0	0	−1	22.44
11	7	−1	0	0	1	22.42
12	21	1	0	0	1	21.10
13	26	0	−1	−1	0	22.04
14	14	0	1	−1	0	21.45
15	18	0	−1	1	0	23.40
16	15	0	1	1	0	26.83
17	23	−1	0	−1	0	25.78
18	10	1	0	−1	0	20.31
19	12	−1	0	1	0	25.19
20	1	1	0	1	0	26.66
21	6	0	−1	0	−1	23.69
22	27	0	1	0	−1	24.79
23	24	0	−1	0	1	20.74
24	5	0	1	0	1	22.94
25	19	0	0	0	0	28.32
26	9	0	0	0	0	30.73
27	2	0	0	0	0	30.59
28	4	0	0	0	0	30.45
29	3	0	0	0	0	29.83

*X*_1_: enzyme concentration (%); *X*_2_: enzymatic time (h); *X*_3_: ultrasonic time (min); *X*_4_: enzymatic temperature (°C).

**Table 4:** Biological sources and activity of the enzyme preparations.

Enzyme Preparations	Biological Source	Activity
CEL	*Trichoderma reseei*	300 U/mg
AXC	*Aspergillus niger*	100 U/mg
GMA	*Trichoderma longibrachiatum*	50 U/mg
PEC	*Aspergillus niger*	500 U/mg
ALC	*Bacillus licheniformis*	200 U/mg
NP	*Bacillus subtilis*	100 U/mg
PAP	*Carica papaya*	800 U/mg

**Table 5:** The variance analysis of the mixed-design model.

Source	df	Sum of Squares	Mean Square	*F*-Value	*p*-Value Prob > *F*
Model	13	482.17	37.09	28.78	0.0003
*x*_1_ *x*_2_	1	10.43	10.43	8.10	0.029
*x*_1_ *x*_3_	1	105.33	105.33	81.74	0.0001
*x*_1_ *x*_4_	1	0.0045	0.0045	0.0035	0.9547
*x*_2_ *x*_3_	1	46.54	46.54	36.12	0.0010
*x*_2_ *x*_4_	1	0.0206	0.0206	0.0160	0.9035
*x*_3_ *x*_4_	1	2.96	2.96	2.30	0.1802
*x*_1_ *x*_2_ *x*_3_	1	0.5306	0.5306	0.4117	0.5448
*x*_1_ *x*_2_ *x*_4_	1	10.71	10.71	8.31	0.0280
*x*_1_ *x*_3_ *x*_4_	1	1.16	1.16	0.8998	0.3795
*x*_2_ *x*_3_ *x*_4_	1	3.17	3.17	2.46	0.1677
Residual	6	7.73	1.29		
Lack-of-fit	1	0.0026	0.0026	0.0017	0.9690
Pure error	5	7.73	1.55		
Total	19	489.90			
R^2^	0.9842				
Adjusted R^2^	0.9500				
C.V%	7.36				

**Table 6:** The variance analysis for the BBD prediction model.

Source	df	Sum of Squares	Mean Square	*F*-Value	*p*-Value Prob > *F*
Model	14	286.9	20.5	24.17	<0.0001
*X*_1_	1	15.4	15.4	18.2	0.0008
*X*_2_	1	10.6	10.5	12.4	0.0033
*X*_3_	1	39.4	39.4	46.4	<0.0001
*X*_4_	1	7.0	7.0	8.2	0.0123
*X*_1_ *X*_2_	1	6.1	6.1	7.2	0.0178
*X*_1_ *X*_3_	1	12.0	12.0	14.2	0.0021
*X*_1_ *X*_4_	1	0.2	0.2	0.2	0.6833
*X*_2_ *X*_3_	1	4.1	4.1	4.8	0.0462
*X*_2_ *X*_4_	1	0.3	0.3	0.4	0.5597
*X*_3_ *X*_4_	1	0.0	0.0	0.0	0.8714
*X*_1_^2^	1	88.5	88.5	104.4	<0.0001
*X*_2_^2^	1	104.4	104.4	123.2	<0.0001
*X*_3_^2^	1	28.1	28.1	33.1	<0.0001
*X*_4_^2^	1	66.8	66.8	78.8	<0.0001
Residual	14	11.9	0.8		
Lack-of-fit	10	7.9	0.8	0.8	0.6440
Pure error	4	3.9	01.0		
Total	28	298.7			
R^2^		0.9603			
Adjusted R^2^		0.9205			
C.V%		3.34			

*X*_1_: enzyme concentration (%); *X*_2_: enzymatic time (h); *X*_3_: ultrasonic time (min); *X*_4_: enzymatic temperature (°C).

## Data Availability

The original contributions presented in the study are included in the article/Supplementary Materials, further inquiries can be directed to the corresponding author.

## Supplementary Materials

The following supporting information can be downloaded at: https://www.mdpi.com/article/10.3390/foods13060891/s1, Figure S1: (A–D) Contour plots of *Ulva* polysaccharide extraction yield (%) calculated from Equation (7); Figure S2: (A–F) Two-dimensional contour plots of the influence of four factors on *Ulva* polysaccharide extraction yield (%).
