# Supplementary material for: Ultrasound-Assisted Multi-Enzyme Extraction for Highly Efficient Extraction of Polysaccharides from Ulva lactuca
Source: Foods. 2024 Mar 15;13(6):891. doi: 10.3390/foods13060891 (PMC10969873; doi:10.3390/foods13060891)
Supplement: Supplementary file 1 [file foods-13-00891-s001.zip › foods-2906669-supplementary.pdf]

Supporting information

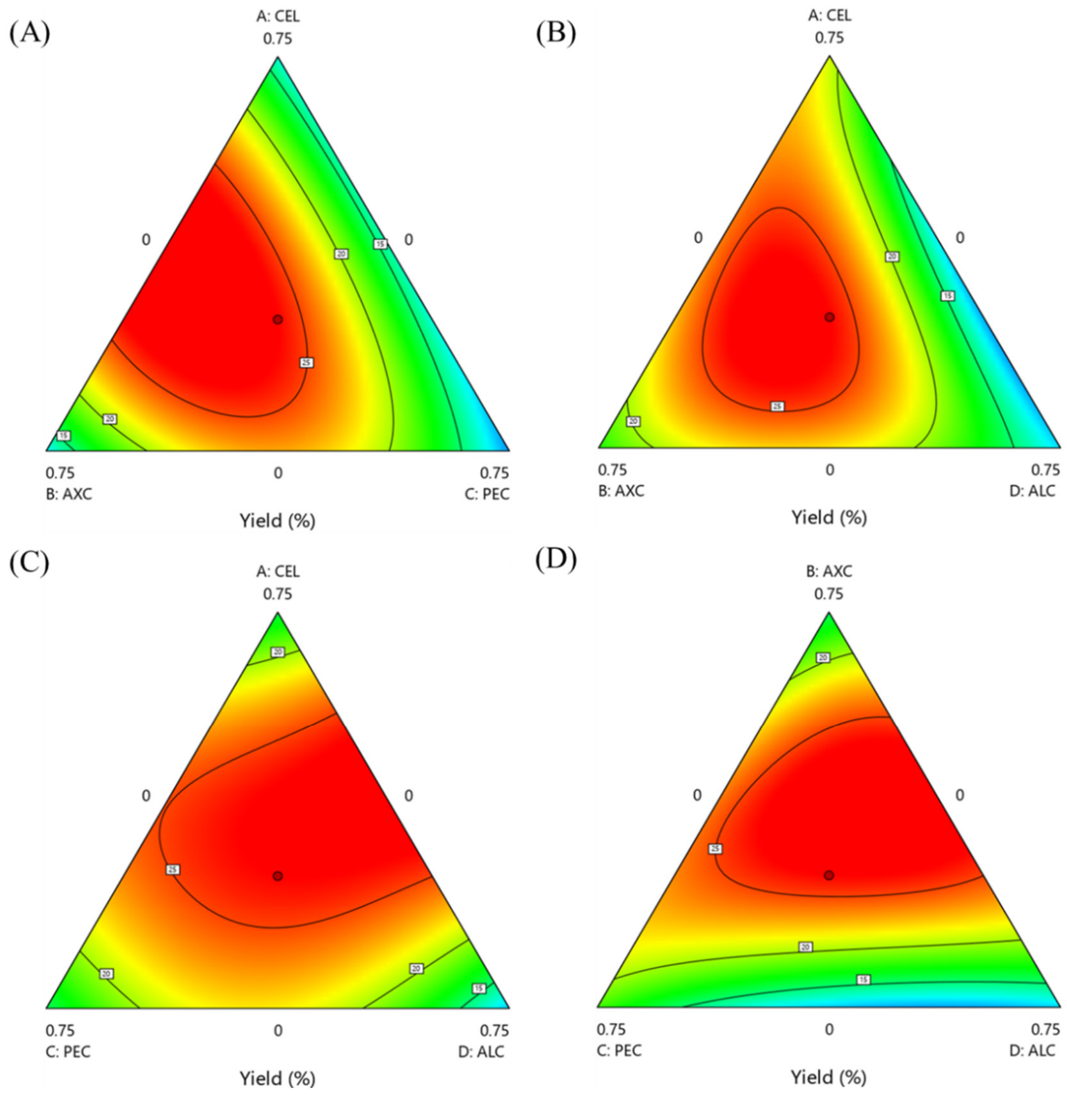

Figure S1 (**A–D**) Contour plots of *Ulva* polysaccharide extraction yield (%) calculated from Eq. 7.

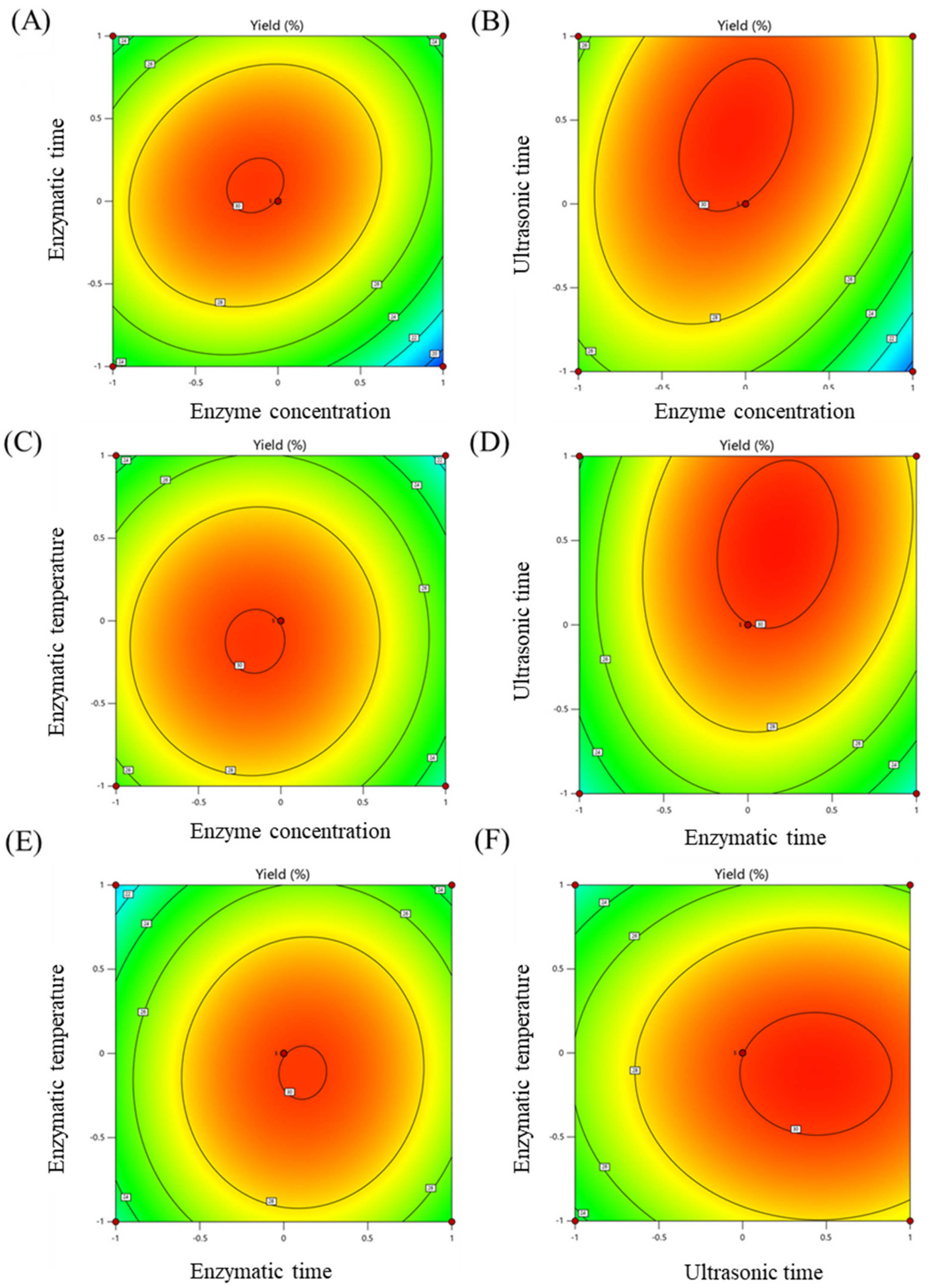

Figure S2 (**A – F**) 2D contour plots of the influence of four factors on *Ulva* polysaccharide extraction yield (%).
